# Use of magnoflorine-phospholipid complex to permeate blood-brain barrier and treat depression in the CUMS animal model

**DOI:** 10.1080/10717544.2019.1616236

**Published:** 2019-05-20

**Authors:** Bingjie Li, Linmeng Han, Bingyan Cao, Xiaoying Yang, Xuehui Zhu, Bing Yang, Haodong Zhao, Wei Qiao

**Affiliations:** a Tianjin Key Laboratory on Technologies Enabling Development of Clinical Therapeutics and Diagnostics, School of Pharmacy, Tianjin Medical University, Tianjin, China;; b Department of Cell Biology, School of Basic Medical Science, Tianjin Medical University, Tianjin, China

**Keywords:** Magnoflorine-phospholipid complex, physicochemical characterization, *in vivo* blood-brain distribution, pharmacological evaluation, western blotting analysis

## Abstract

To improve the liposolubility and blood-brain barrier permeability of magnoflorine, a new formulation of magnoflorine-phospholipid complex was prepared, characterized, and pharmacologically evaluated in the chronic unpredictable mild stress animal model. In this paper, the magnoflorine-phospholipid complex was synthesized and its characterization was determined. The antidepressant-like and antioxidant activity of magnoflorine-phospholipid complex was investigated by behavioral tests and western blotting analysis. As a result, the magnoflorine-phospholipid complex displayed high encapsulation efficiency and significantly improved the oil-water participate coefficient. *In vivo* blood-brain distribution study, the magnoflorine-phospholipid complex extended the duration of magnoflorine in blood and help magnoflorine to permeate the blood-brain barrier into brain. In behavioral tests, the magnoflorine-phospholipid complex significantly decreased immobility time compared to model control group in both FST and TST. Furthermore, the magnoflorine-phospholipid complex increased the expression of antioxidative stress-related proteins by the western blotting analysis. These findings strongly suggest that the phospholipid complex could significantly improve liposolubility, drug properties of magnoflorine and help magnoflorine permeate blood-brain barrier and exert the antidepressant effect.

## 1. Introduction

Drug design and development is an important aspect of a novel drug delivery system. The rate and extent of the antipsychotic medications (the central nervous system-acting drugs) permeating the membranes of intestinal tract or brain into the bloodstream or cerebrospinal fluid depends on solubility in lipid, which plays a crucial role in determining transport properties of small or large drug molecules and affects the pharmacokinetics, especially drug absorption, diffusion, and release (Van Nijlen et al., 2003; Nokhodchi et al., 2005; Zhang, Zhang, et al., 2016). *Ziziphi spinosae semen* (ZSS, *suanzaoren* in Chinese) is the dried and ripe seeds of *Ziziphus jujuba* Mill. *var. spinosa* (Bunge) Hu ex H. F. Chou. As a popular herb used long in China, ZSS has the functions of supplementing the liver, quieting the heart, arresting the sweat, and promoting the production of the body fluid from views of Traditional Chinese Medicine. It is the most frequently used herb in Chinese materia medica preparations to treat anxiety and insomnia for its main sedative and hypnotic effects (Sun et al., 2014; Zhang, Li, et al., 2016). Up till now, phytochemical studies on ZSS have resulted in the isolation of flavonoids (Cheng et al., 2000; Zhang et al., 2012), saponins (Wang et al., 2013), alkaloids (Han et al., 1989; Park et al., 1996), terpenes, as well as a series of indole derivatives (Li et al., 2014). Magnoflorine is one of the main alkaloids component in the ZSS. In the previous study, magnoflorine was discovered of its good function on protecting the cardiovascular system (Chen et al., 1990), regulating immune function (Xie et al., 2011), and antioxidation (Peng et al., 1995). Recently, our study firstly found that magnoflorine had good sedative-hypnotic and antidepressant function. But due to its bad liposolubility, which caused its low permeability and bioavailability, the ideal therapeutic effect of magnoflorine is severely influenced. Therefore, improving the liposolubility is the key for magnoflorine to increase its permeability and bioavailability.

Phospholipid complex is a novel drug delivery system in recent years. As an amphoteric material, phospholipid has the polar groups (hydrophilic part) and long fatty acid chain (lipophilic part). So phospholipid can not only improve the hydrophilicity of drugs, but also increase the lipophilicity of drugs (Zhang et al., 2015). Many natural drugs, especially some types of flavonoids, may exhibit a marked affinity to phospholipids (Telange et al., 2016; Zhang, Zhang, Zhang, 2016). The drug-phospholipid complex, so-called phytosomes, show some superior chemicophysical properties and enhance the permeability of drugs. One recent study with repaglinide-phospholipid complex showed that the formulation promoted the antidiabetic of repaglinide in an alloxan-induced diabetic rat model (Kassem et al., 2017). It was found that saponin-phospholipid complex exerted better anticancer effects than a pure molecule in rats (Dang Kim et al., 2016).

In this study, magnoflorine-phospholipid complex was prepared to improve its lipophilicity, permeability, and bioavailability. The study focused on the preparation, characterization, and pharmacological evaluation of magnoflorine-phospholipid complex.

## 2. Materials and methods

### 2.1. Materials and instrumentations

#### 2.1.1. Materials

Magnoflorine was made by Tianjin Key Laboratory on Technologies Enabling Development of Clinical Therapeutics and Diagnostics (Tianjin Medical University, China, Purity >95%). The phospholipid was purchased from Lanji (Lanji Biological, Shanghai, China). Amitriptyline (95%) hydrochloride was purchased from Dongting (Hunan Dongting Pharmaceutical, Hunan, China). Ethanol was purchased from Concord (Concord Technological, Tianjin, China). Dichloromethane and *n*-butyl alcohol were obtained from Benchmark (Benchmark Chemical, Tianjin, China). All other reagents were of analytical grade.

#### 2.1.2. Instrumentations

Agilent 1260 high-performance liquid chromatography (HPLC) was purchased from Agilent (California, USA), HT7700 transmission electron microscopy (TEM) was purchased from Hitachi (Tokyo, Japan), 214 polyma differential scanning calorimeter (DSC) was purchased from Netzsch (Selbe, Germany), Nicolet 380 fourier transform infrared spectroscopy was purchased from Hitachi (Tokyo, Japan), Delsa Nano size analyzer was purchased from Beckman Coulter (Kraemer Boulevard Brea, USA), ZH-YLS-1A animal locomotor actimeter was purchased from Zhenghua Biologic Apparatus facilities company (Anhui, China).

### 2.2. Animals

Male ICR mice (20 ± 2 g) were obtained from Experiment Animal Center of Chinese Academy of Medical Sciences (Tianjin, China). Animals were housed in the laboratory conditions at room temperature (22 ± 2 °C), relative humidity (65%–70%) with 12 h light/dark cycles. They acclimated to 3 days before experiments and meet ethical requirements.

### 2.3. Blood-brain barrier permeability prediction of magnoflorine

Blood-brain barrier permeability of magnoflorine was predicted from different basic properties (H Bonds, MW, pka_1_, N + O, TPSA, Log D, Clog P) by software ACD/I Lab (for Canada).

### 2.4. Preparation of the magnoflorine-phospholipid complex

The complex was prepared with magnoflorine and phospholipid at a molar ratio of 1:2. Both the reactants were taken in a 50 mL round bottom flask and 20 mL of ethanol was added. The reaction was proceeded by refluxing the reaction mixture in a magnetic stirrer at 120 r/min, 55 °C for 2 h. After reaction, the ethanol used as solvent was removed and the sufficient amount of dichloromethane was added to get precipitation. Then the magnoflorine-phospholipid complex was collected, washed, and dried under vacuum to remove the dichloromethane. Finally, the complex was stored in an airtight glass bottle at room temperature until further use.

### 2.5. Encapsulation efficiency of the magnoflorine-phospholipid complex

Magnoflorine-phospholipid complex (50 mg, without precipitation) and 1 mL dichloromethane was placed in a 2 mL centrifuge tube, ultrasonic dissolution, and centrifuged for 10 min at 5000 r/min. Then the undissolving substance was collected, washed, and dried under vacuum. The amount of magnoflorine in the undissolving substance was determined by Agilent 1260 HPLC. Encapsulation efficiency was calculated by using the formula:

Encapsulation efficiency (%) = (A−B)/A×100%

where A is magnoflorine content in the magnoflorine-phospholipid complex (without precipitation), B is magnoflorine content in the undissolving substance.

### 2.6. DSC of the magnoflorine-phospholipid complex

The DSC curve of magnoflorine, phospholipid, physical mixture of magnoflorine and phospholipid, and magnoflorine-phospholipid complex were obtained on the 214 polyma DSC. The samples were sealed in the aluminum crucible and heated at the rate of 10 °C/min from 0 to 300 °C under nitrogen gas flow.

### 2.7. Infrared spectrum (IR) of the magnoflorine-phospholipid complex

The infrared spectrum (wavelength range: 400–4000 cm^−1^) of magnoflorine, phospholipid, physical mixture of magnoflorine and phospholipid, and magnoflorine-phospholipid complex were recorded on a Nicolet 380 fourier transform infrared spectroscopy with pure KBr as the background.

### 2.8. TEM of the magnoflorine-phospholipid complex

The morphology and microstructure of the magnoflorine-phospholipid complex were characterized by an HT7700 TEM. The sample for TEM was prepared by drying complex droplets from water dispersion onto a 300-mesh Cu grid coated with a lacey carbon film.

### 2.9. Oil-water participate coefficient (P) study

Magnoflorine solution and the magnoflorine-phospholipid complex solution were prepared by dispersing magnoflorine or the complex in the *n*-butyl alcohol saturated with water. The oil-water participate coefficient was determined by adding 1 mL magnoflorine solution or the complex solution to 4 mL hydrochloric acid solution (pH 1.2), different phosphate buffer solution (pH 2.0, 2.5, 4.0, 5.8, 6.8, 7.4, 8.0) or water saturated with *n*-butyl alcohol in the centrifuge tube. Then the mixtures were shaken in the thermostat oscillator at 37 °C for 24 h and centrifuged for 30 min at 3000 r/min. The aqueous solution was collected, diluted, and filtered through a membrane filter (0.45 μm). Finally, these samples were measured by Agilent 1260 HPLC. The oil-water participate coefficient was calculated by using the formula:

$$P\;=\;\left(W_{\text{1}}/V_{\text{a}}\right)/\left(W_{\text{2}}/V_{\text{b}}\right)$$

where *P* is oil-water participate coefficient of magnoflorine, *W*_1_ is magnoflorine content in the oil solution, *W*_2_ is magnoflorine content in the water solution, *V*_a_ is the volume of the oil solution, *V*_b_ is the volume of the water solution.

### 2.10. In vivo blood-brain distribution study

#### 2.10.1. Animals and dosing

After 3 days of acclimatization, male ICR mice were randomly divided into 8 groups. Group 1 and Group 2 were given approximately the same volume of water and served as normal groups. Group 3 and Group 4 were given magnoflorine (17 mg·kg^−1^) and served as magnoflorine control groups. Group 5 and Group 6 were given magnoflorine-phospholipid complex (equivalent to magnoflorine 17 mg·kg^−1^). Group 7 and Group 8 were given approximately the same volume of phospholipid and served as model groups. The mice of odd groups were fasted for 12 h, blood samples and brain samples were collected at 5 min post intravenous injection. The mice of even groups were fasted for 12 h, blood samples and brain samples were collected at 30 min post intravenous injection.

#### 2.10.2. Preparation of blood serum sample and brain extract sample

Blood samples stand for 30 min, centrifuged at 5000 r/min for 10 min and blood serum samples were collected. PBS solution was added to grind brain samples, abrasives centrifuged at 5000 r/min for 10 min and brain extract samples were collected. Then these samples were vortex-mixed with the same volume of methanol for 5 min, repeatedly centrifuged at 5000 r/min for 10 min. After centrifugation, the supernatant was evaporated to dryness. The obtained residues were dissolved in 0.5 mL methanol and filtered through a membrane filter (0.45 μm). Finally, these samples were measured by Agilent 1260 HPLC.

### 2.11. Antidepressant activity of the complex in mice

#### 2.11.1. Animals and dosing

After 3 days of acclimatization, male ICR mice were randomly divided into 4 groups. Group 1 was given approximately the same volume of water and served as a normal group. Group 2 was given approximately the same volume of water and served as model control group. Group 3 was given amitriptyline hydrochloride (15.03 mg·kg^−1^) and served as positive control group. Group 4 was given magnoflorine-phospholipid complex (equivalent to magnoflorine 7 mg·kg^−1^).

In addition to the normal group, other groups were subjected to the chronic unpredictable mild stress (CUMS) animal model of depression for 21 days (Ishikawa et al., 2017) and exposed daily to two of the following stressors in a random order: 45°cage tilting, wet bedding, no bedding, swimming in hot water, swimming in cold water, food deprivation, water deprivation, clipping tails, 12 h lights on, and 12 h lights off.

#### 2.11.2. Behavioral tests

##### 2.11.2.1. Forced swim test

The forced swim test (FST) was used to assess behavioral despair as a rodent test for screening antidepressant activity of drugs. Each mouse was individually placed in a glass cylinder (20 cm diameter, 32 cm height) containing water (25 ± 1 °C) up to 20 cm. When the mouse did not struggle and made only movements necessary to keep its head above water, the immobility time was recorded during 6 min as a depressive-like state. Immobility time was presented during the last 4 min of the swimming session. After each session, the water was changed and the animals were dried and replaced in their home cage (Marques et al., 2017).

##### 2.11.2.2. Tail suspension test

Mice were suspended by their tails with tape, and when the mouse stopped struggling and hanged immobile, the immobility time during a 6 min session was measured as a depressive-like state. Immobility time was presented during the last 4 min of the suspension session (Nikoui et al., 2016).

#### 2.11.3. Western blot analysis

Mice were killed 30 min later after the last administration and brains were collected. Then, PMSF RAPA lysate was added to crack brain tissue, centrifuged at 4 °C 12,000 r/min for 20 min, collected protein samples and stored at −80 °C until use.

In order to assess the effects of magnoflorine-phospholipid complex on the oxidative stress response, the expression level of four antioxidative related proteins (NRF2, Keap1, NQ, HO-1) was examined via western blotting. Briefly, 20 μL protein samples were mixed with SDS buffer solution and separated on a 15% (v/v) polyacrylamide gel by 80 V electrophoresis. The separated proteins were transferred electrically (0.3 A for 2 h) to a polyvinylidene fluoride (PVDF) membrane. The membranes were blocked in 5% (w/v) skim milk in Tris-buffered saline-Tween 20 (TBST) and then incubated with the primary antibodies for NRF2, Keap1, NQ, and HO-1, respectively. The membranes were washed three times with TBST and incubated with the secondary antibodies. After three further washes in TBST, the membranes were exposed to western blotting reagents and the signals were detected by a luminescence imaging system.

### 2.12. Statistical analysis

Analyses of data were carried out using GraphPad Prism 6 software (for San Diego). For multiple comparisons, one-way analysis of variance (ANOVA) was used. Results were expressed as mean ± S.E.M. *p* values less than .05 were considered statistically significant.

## 3. Results and discussion

### 3.1. Blood-brain barrier permeability prediction of magnoflorine

From Clark’s rules and Pardridge’s rules, if a compound can permeate the blood-brain barrier, it should satisfy the following conditions at the same time, concluding H Bonds﹤8–10, MW﹤450, nonacid compound, N + O ﹤6, TPSA﹤60–70Å^2^, log D = 1–3, Clog P−(N + O)>0. As shown in Table 1, because of log D ≠ 1–3 and Clog P−(N + O) <0, blood-brain barrier permeability of magnoflorine is low, leading to hardly permeating the blood-brain barrier.

**Table 1.** Blood-brain barrier permeability prediction of magnoflorine.

Properties	H bonds	MW	pka_1_	N + O	TPSA	Log D	Clog P−(N + O)
Magnoflorine	7	342.41	9.23	4	58.92	−3.03	−5.71

### 3.2. Encapsulation efficiency of the complex

Encapsulation efficiency of the magnoflorine-phospholipid complex was determined by HPLC method. In this study, we found that magnoflorine has good encapsulation efficiency in the phospholipid complex and yielded 99% (w/w) as estimated by Agilent 1260 HPLC.

### 3.3. DSC of the complex

**Figure 1.**
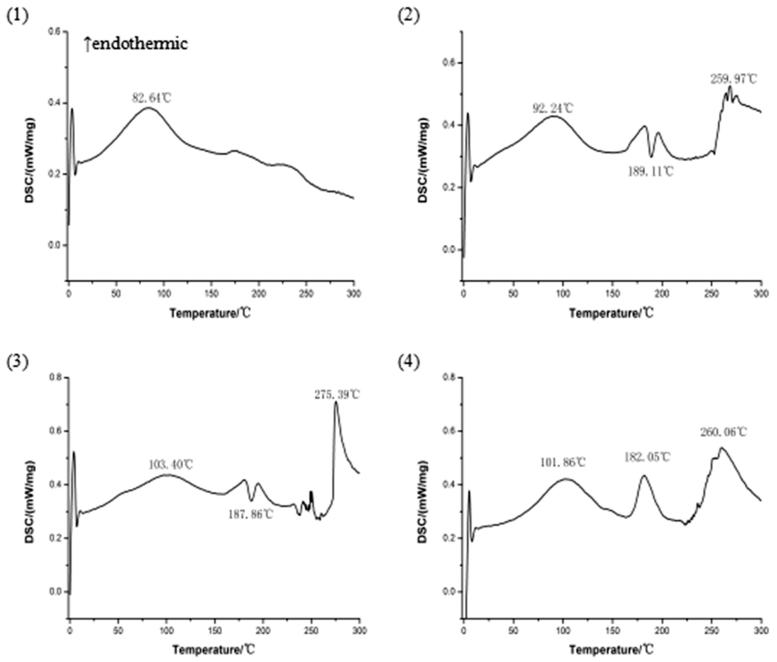
The DSC curves: (1) magnoflorine, (2) phospholipid, (3) physical mixture of magnoflorine and phospholipid, (4) magnoflorine-phospholipid complex.

DSC is a fast and reliable method to screen drug-excipient compatibility and provides maximum information about the possible interactions (Meng et al., 2016). In DSC, an interaction is concluded by the elimination of exothermic peak(s), appearance of new peak(s), change in peak shape and its onset, peak temperature/melting point and relative peak area or enthalpy (Maiti et al., 2007). The DSC curves of magnoflorine, phospholipid, physical mixture of magnoflorine and phospholipid, and magnoflorine-phospholipid complex are shown in Figure 1. The DSC curve of magnoflorine exhibited an endothermic peak at 82.64 °C, caused by the melting of magnoflorine. Phospholipid exhibited two endothermic peaks and an exothermic peak. The first endothermic peak was at 92.24 °C, which appeared due to the hot movement of phospholipid polar head group and the second one at 259.97 °C. And phospholipid exhibited a sharp exothermic peak at 189.11 °C, which appeared due to phase transition from solid to liquid state. The physical mixture of magnoflorine and phospholipid also exhibited two endothermic peaks and an exothermic peak. The first endothermic peak at 103.4 °C appeared because of superposition between an endothermic peak of magnoflorine (82.64 °C) and one of phospholipid (92.24 °C).The second endothermic peak at 275.39 °C appeared due to phase transition from gel to liquid crystalline state. The second endothermic peak (275.39 °C) and exothermic peak (187.86 °C) have a similar onset temperature of phospholipid. A sharp new endothermic peak, with an onset temperature at 182.05 °C, appeared in the DSC curve of the magnoflorine-phospholipid complex, which is different from the peaks of the individual components of the complex. Therefore, it is possible that magnoflorine can interact with phospholipid and form the strong force which may need enough energy to break.

### 3.4. Infrared spectrum (IR) of the complex

**Figure 2.**
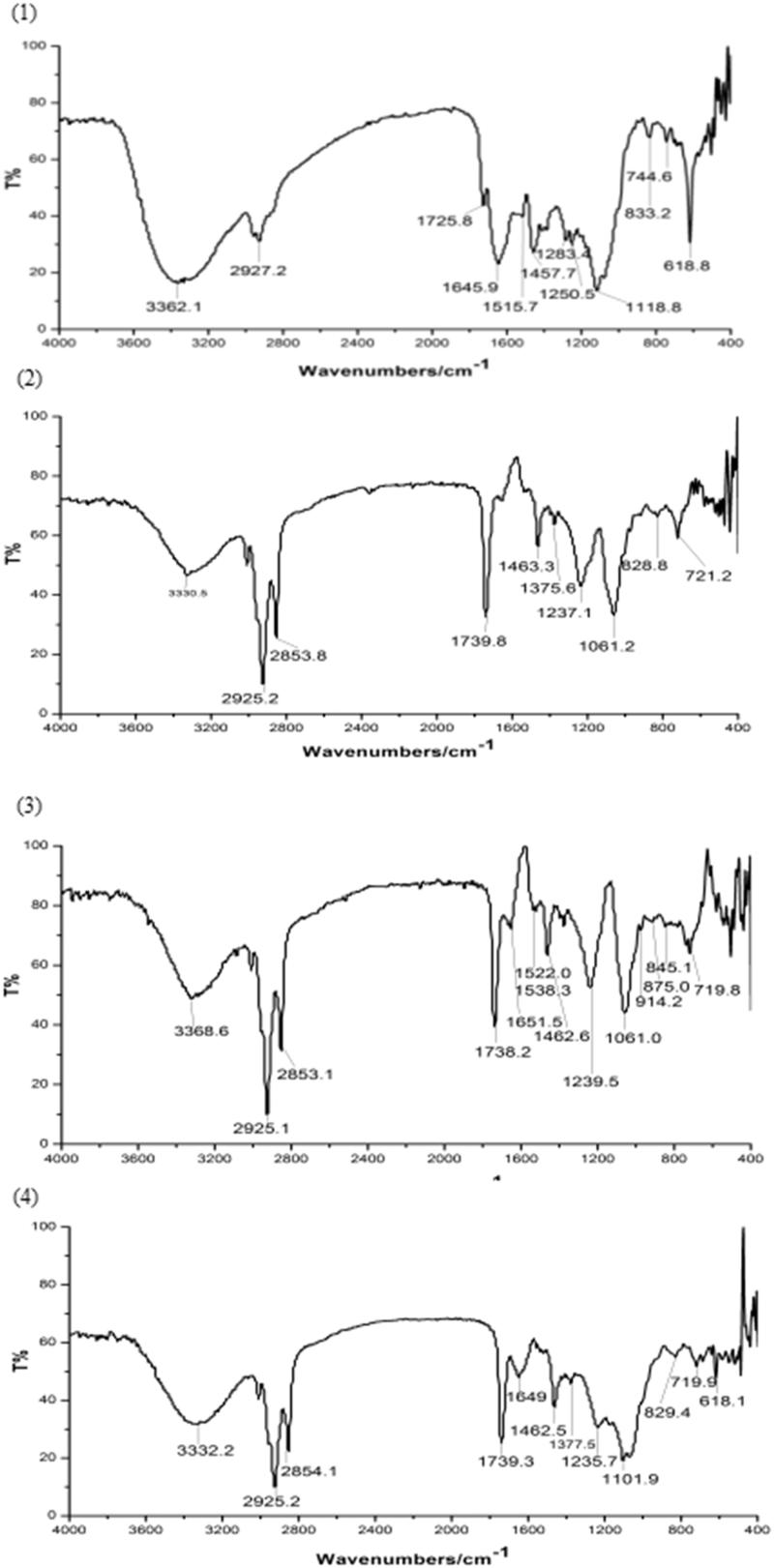
The IR spectra: (1) magnoflorine, (2) phospholipid, (3) physical mixture of magnoflorine and phospholipid, (4) magnoflorine-phospholipid complex.

Infrared spectrum studies are done to detect the possible interaction between magnoflorine and phospholipid in the phospholipid complex (Singh et al., 2013). The IR spectra of magnoflorine, phospholipid, physical mixture of magnoflorine and phospholipid, and magnoflorine-phospholipid complex are shown in Figure 2. The IR spectrum of magnoflorine showed a characteristic peak at 3362.1 cm^−1^, which could be attributed to O–H stretching. 1645.9 cm^−1^ and 1515.7 cm^−1^ were ascribed C = C stretching band of benzene. Furthermore, significant peak could be measured at 1283.4 cm^−1^ and 1250.5 cm^−1^, which were related to C–O stretching in magnoflorine structure (Ebrahimi et al., 2015). The IR spectrum of phospholipid showed characteristic peaks at 2925.2 cm^−1^ and 2853.8 cm^−1^, which could be attributed to C–H stretching band of long fatty acid chain. The C = O stretching band of fatty acid ester was detected at 1739.8 cm^−1^, while a peak at 1237.1 cm^−1^ was probably due to P = O stretching (Rawat et al., 2013). Characteristic peaks at 1463.3 cm^−1^ and 1375.6 cm^−1^ represented C–H stretching of methyl. Another peak at 1061.2 cm^−1^ was ascribed to P–O–C stretching. The IR spectrum of physical mixture of magnoflorine and phospholipid also exhibited the characteristic peaks of magnoflorine and phospholipid, and showed apparently as the superposition of magnoflorine and phospholipid spectrum. It suggested that magnoflorine would have not any interaction with phospholipid in the physical mixture of magnoflorine and phospholipid. In the IR spectrum of magnoflorine-phophoslipid complex, significant changes were observed. The characteristic peaks of O–H stretching of magnoflorine shifted to lower wave number (3332.2 cm^−1^) and the characteristic peaks of C–O stretching were completely disappeared, which suggested possible interaction sites with phospholipid. The characteristic peaks of C = C stretching band of benzene changed and peak at 1515.7 cm^−1^ was completely disappeared. In addition, the characteristic peaks of P–O–C stretching of phospholipid shifted to new wave number (1104.1 cm^−1^). But the characteristic peaks of long fatty acid chain of phospholipid were not changed. These spectroscopic changes give an evidence of the existence of interaction between oxygen negative ion of magnoflorine and nitrogen positive ion of polar end of phospholipid and complex formation.

### 3.5. TEM of the complex

**Figure 3.**
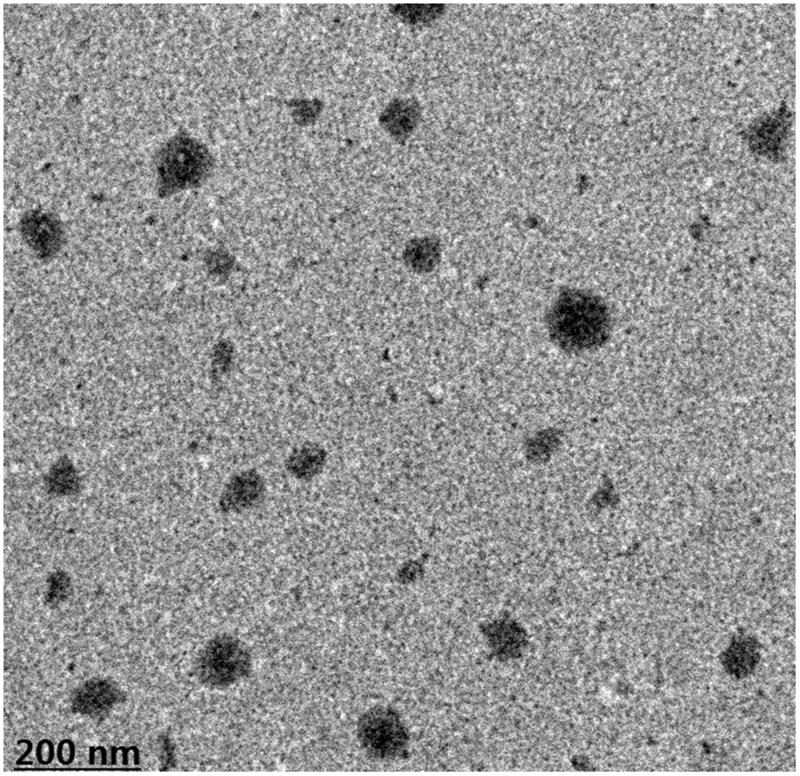
TEM micrograph of the magnoflorine-phospholipid complex.

The morphology and microstructure of the magnoflorine-phospholipid complex are shown in Figure 3. The transmission electron microscopic photograph of the complex indicated that the magnoflorine-phospholipid complex was similarly fluffy spherical particles in the water, even dispersing. And the structure of a good size was about 200 nm or so. Magnoflorine was intercalated in the lipid layer and the hydrophobic chain of the phospholipid was free around the particle to improve the drug lipotropy.

### 3.6. Oil-water participate coefficient (P) study

**Figure 4.**
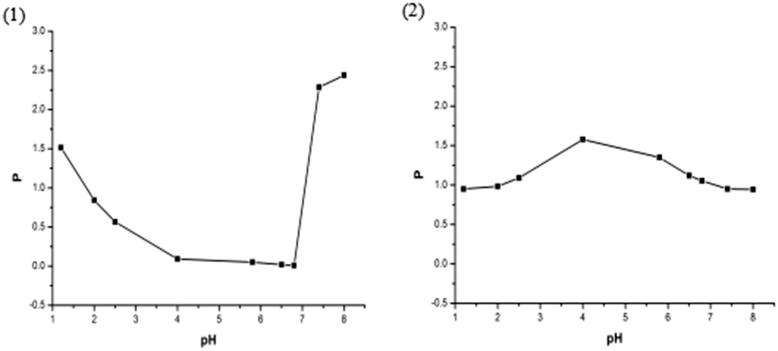
The oil-water participate coefficient of magnoflorine curves from (1) free magnoflorine and (2) magnoflorine-phospholipid complex in different pH.

The oil-water participate coefficient curves of magnoflorine from free magnoflorine and magnoflorine-phospholipid complex are shown in Figure 4. From the oil-water participate coefficient curve of magnoflorine, we found that the average oil-water participate coefficient of magnoflorine is 0.868 and it shows a poor solubility in the *n*-butyl alcohol solution. So that magnoflorine exhibits low bioavailability *in vivo* pharmacology experiment because a drug must permeate the membranes of brain or intestinal tract before it can reach the brain or systemic circulation to exert effect (Hancock & Zografi, 1997; Semalty et al., 2009). When magnoflorine was combined with phospholipid, the complex had a higher average oil-water participate coefficient (1.113) than free magnoflorine. And it improved the solubility of magnoflorine in the *n*-butyl alcohol solution significantly, which may be explained by the amorphous characteristics of the complex (due to reduced molecular crystallinity of the drug) and amphiphilic nature of the complex (Lichtenberger et al., 1996; Yanyu et al., 2006). Therefore, the good oil-water participate coefficient and high liposolubility of magnoflorine-phospholipid complex would give a positive impact on the absorption and bioavailability of the drug (Blagden et al., 2007; Ambrus et al., 2009).

### 3.7. In vivo blood-brain distribution study

**Figure 5.**
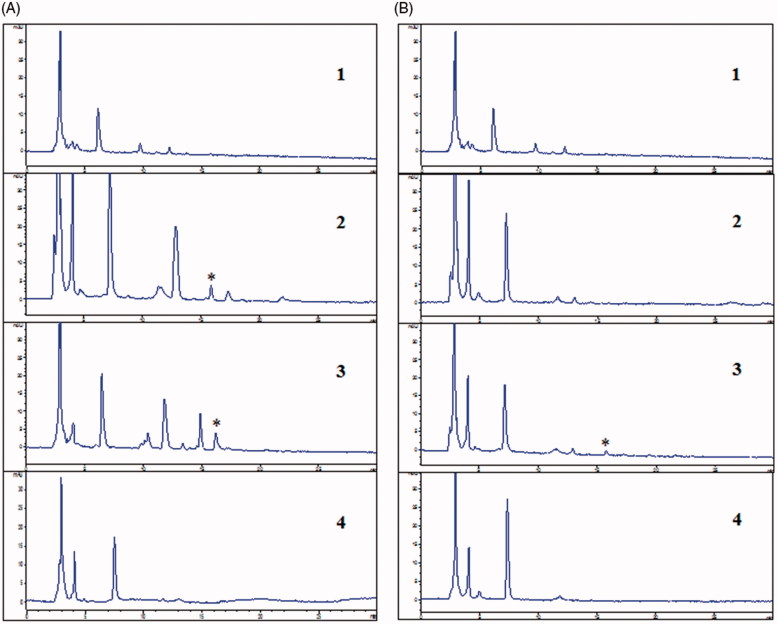
HPLC chromatograms of 5 min blood serum (A) and 30 min blood serum (B): (1) normal, (2) magnoflorine, (3) magnoflorine-phospholipid complex, (4) phospholipid. *Magnoflorine chromatographic peak.

In order to verify the brain targeting of magnoflorine-phospholipid complex, *in vivo* blood-brain distribution study was performed in male ICR mice. As shown in Figure 5(A), at 5 min post intravenous injection, we found that the significant magnoflorine chromatographic peak at 16 min appeared in blood serum of magnoflorine control group and magnoflorine-phospholipid complex group. But there was no any chromatographic peak at 16 min appeared in blood serum of normal group and model group. It suggested that endogenous ingredients of blood serum and phospholipid did not interfere magnoflorine chromatographic peak. As shown in Figure 5(B), at 30 min post intravenous injection, we found that the significantly lower magnoflorine chromatographic peak at 16 min appeared in blood serum of magnoflorine-phospholipid complex group than that in 5 min blood serum of magnoflorine-phospholipid complex group. This explained that the content of magnoflorine significantly decreased from 5 min to 30 min post intravenous injection. But there was no magnoflorine chromatographic peak appeared in blood serum of magnoflorine control group. It suggested that magnoflorine may be completely metabolized in blood serum at 30 min post intravenous injection. On the other hand, it showed that the complex extended the duration of magnoflorine in blood and had sustained release effect.

As shown in Supplementary Figure 6(C), at 5 min post intravenous injection, we found that the small magnoflorine chromatographic peak at 16 min appeared in brain extract of magnoflorine-phospholipid complex group. But there was no magnoflorine chromatographic peak appeared in brain extract of magnoflorine control group. It suggested that magnoflorine of the phospholipid complex may start to permeate the blood-brain barrier into brain at 5 min post intravenous injection. As shown in Supplementary Figure 7(B), at 30 min post intravenous injection, we found that the significantly higher magnoflorine chromatographic peak at 16 min appeared in brain extract of magnoflorine-phospholipid complex group than that in 5 min brain extract of magnoflorine-phospholipid complex group. This explained that the content of magnoflorine significantly increased from 5 min to 30 min post intravenous injection. Supplementary Figure 6(D), confirmed that magnoflorine of the phospholipid complex indeed could permeate the blood-brain barrier into brain to exert effect at 30 min post intravenous injection and the phospholipid complex significantly improved blood-brain barrier permeability of magnoflorine.

### 3.8. Antidepressant activity of the complex in mice

#### 3.8.1. Effect of the complex on behavioral tests

In forced swim test, magnoflorine and magnoflorine-phospholipid complex group compared with model control group, both mice FST immobility time were significantly shorter, have statistically significant (*p* < .01), but magnoflorine-phospholipid was more effective than magnoflorine at the same dose. It is suggested that magnoflorine-phospholipid complex showed a significant antidepressant-like response.

In tail suspension test, magnoflorine and magnoflorine-phospholipid complex group compared with model control group, both mice TST immobility time were significantly shorter, have statistically significant (*p* < .05), and both effects were similar at the same dose. It is further confirmed that magnoflorine-phospholipid complex exerted a significant antidepressant effect.

#### 3.8.2. Western blot analysis

Oxidative stress refers to the body being subjected to various stimuli, resulting in increased levels of reactive oxygen in the body. Exceeding the elimination level of the body, the body’s oxidation system and the antioxidant system are out of balance, causing a series of damage to the body. According to studies, the imbalance of the oxidation system is the pathophysiological basis of many diseases, such as depression (Diniz et al., 2018). Nrf2 (Nuclear factor erythroid-2 related factor 2) is a key factor in cellular oxidative stress response and regulated by Keap1. Through the interaction with antioxidant response element ARE, Nrf2 regulates the expression of antioxidant protein and phase II detoxification enzyme (NQ, HO-1).

As shown in Supplementary Figure 9, magnoflorine-phospholipid complex group compared with model control group, the expression of three antioxidative stress-related proteins (Nrf2, NQ, HO-1) was significantly increased. It indicated that the magnoflorine-phospholipid complex exhibited antioxidant effect and protected neurons via the Nrf2/ARE pathway.

## 4. Conclusions

In the study, because of low blood-brain barrier permeability of magnoflorine predicted by virtual screening, so that the ideal antidepressant effect is severely influenced. Therefore, a new formulation (magnoflorine-phospholipid complex) is expected to improve lipophilicity, blood-brain barrier permeability, and bioavailability of magnoflorine. The present study successfully prepared magnoflorine-phospholipid complex by a simple and reproducible method. Based on DSC and infrared spectrum data, the resultant complex was confirmed. There was significantly higher oil-water participate coefficient of magnoflorine from magnoflorine-phospholipid complex than from free magnoflorine. The improved liposolubility and lipophilicity indicated that the complex may be easier to permeate the membranes of brain into the brain to exert an effect. Then, from *in vivo* blood-brain distribution study, it was confirmed that the complex significantly improved blood-brain barrier permeability of magnoflorine and help magnoflorine into brain, which could be a benefit to exert the antidepressant effect. Finally, from behavioral tests and western blot analysis, the results further confirmed that magnoflorine-phospholipid complex had significant antidepressant and antioxidant effects.

## Supplementary Material

supplementary.docx
