# Supplementary figures and images for: Use of magnoflorine-phospholipid complex to permeate blood-brain barrier and treat depression in the CUMS animal model
Source: Drug Deliv. 2019 May 20;26(1):566–74. doi: 10.1080/10717544.2019.1616236 (PMC6534223; doi:10.1080/10717544.2019.1616236)

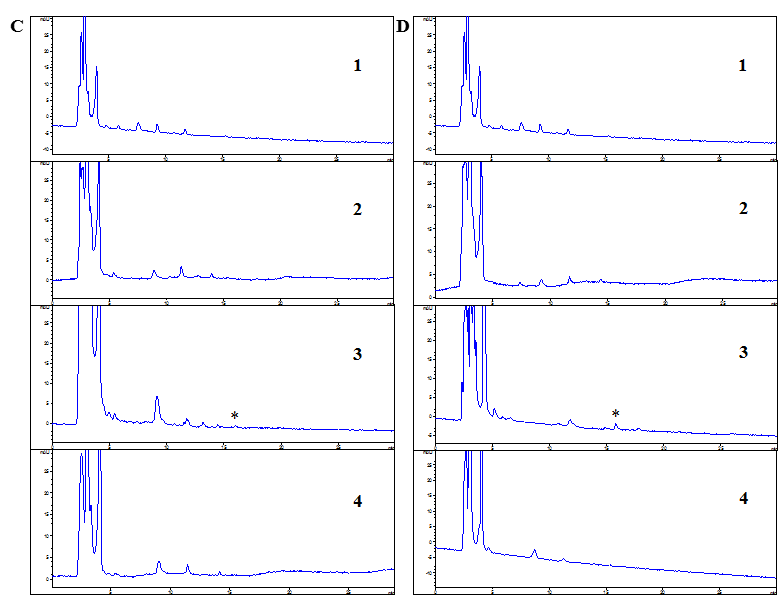
Fig.6


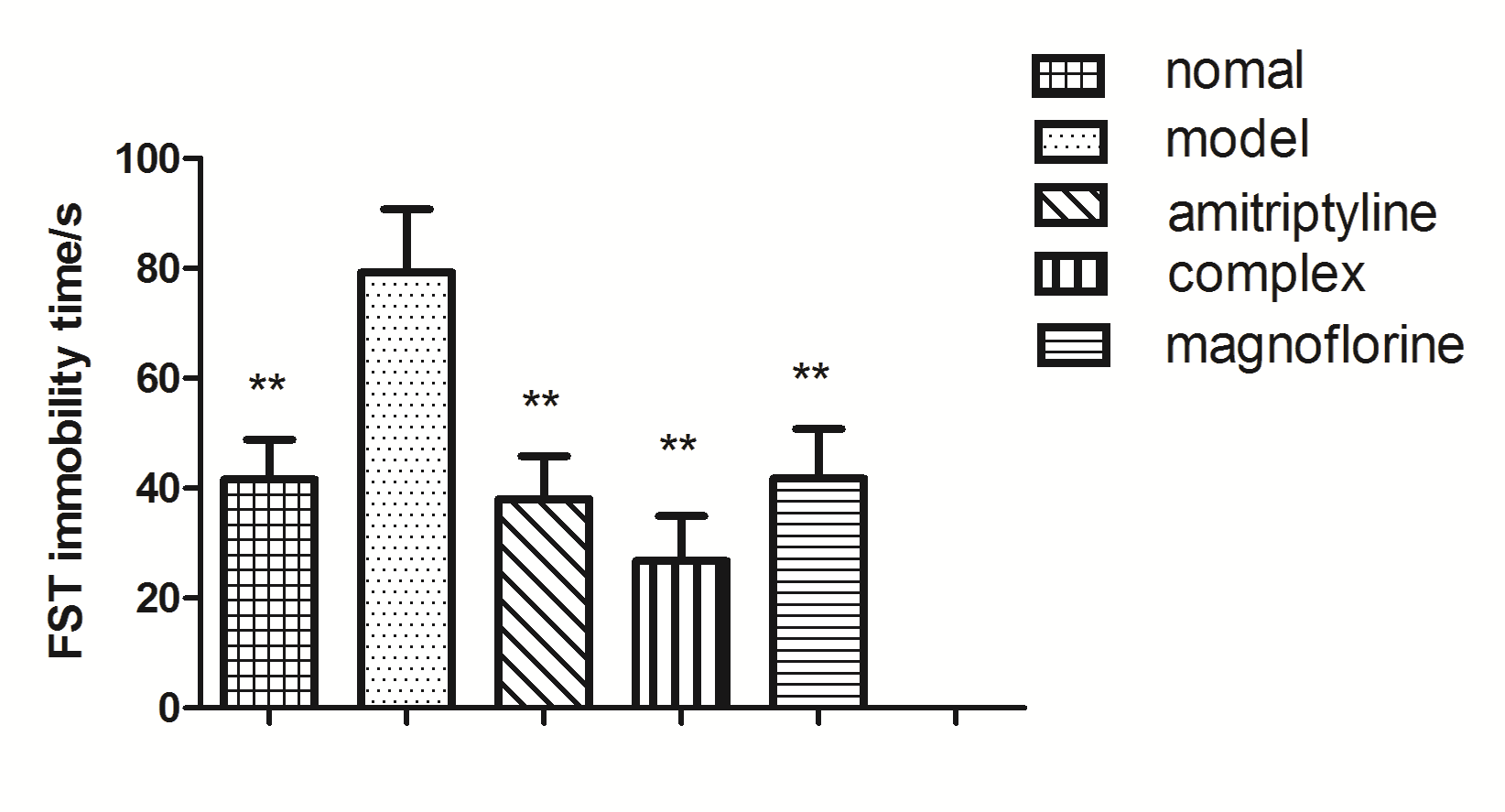


Fig.7


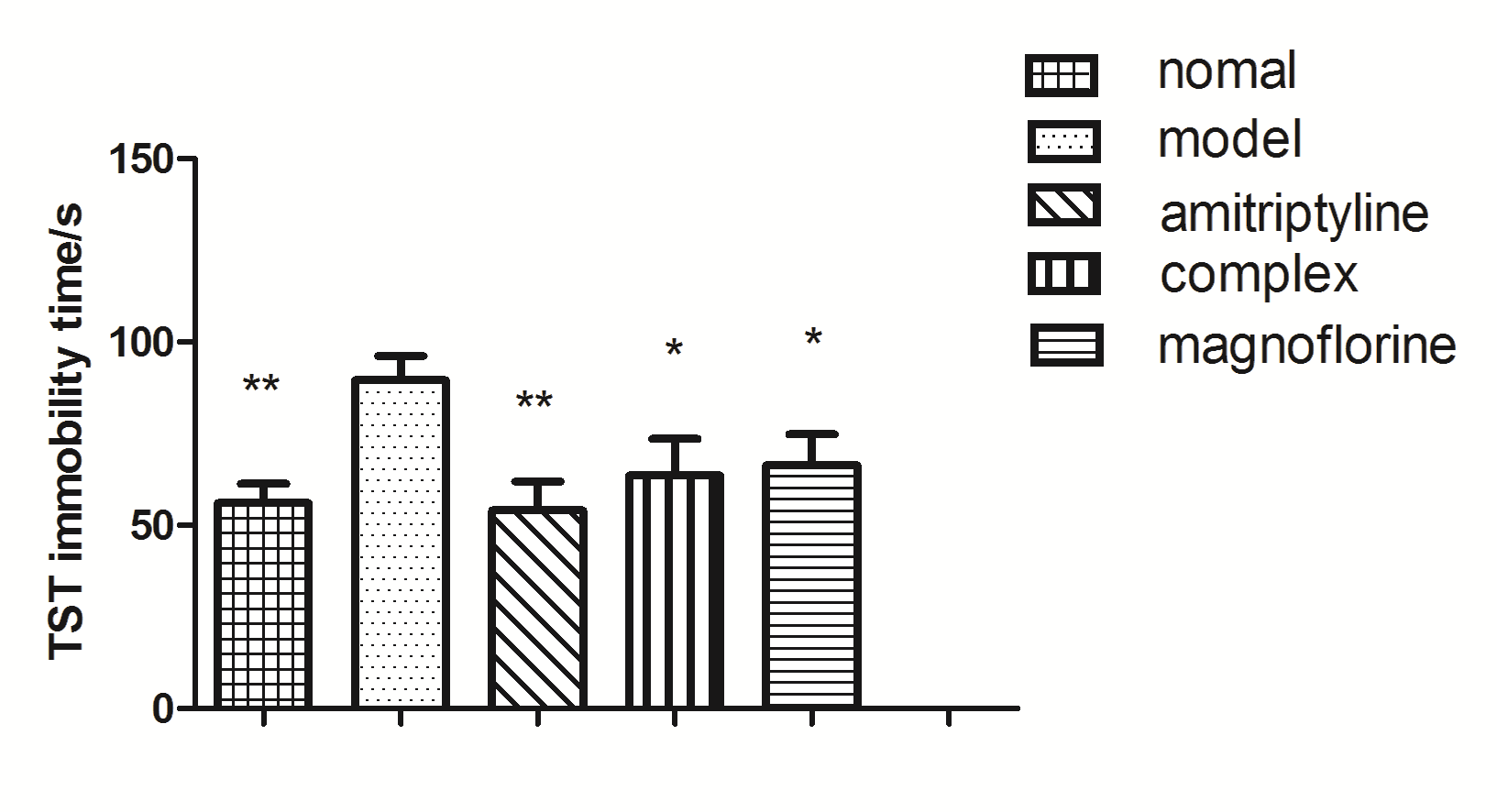


Fig.8


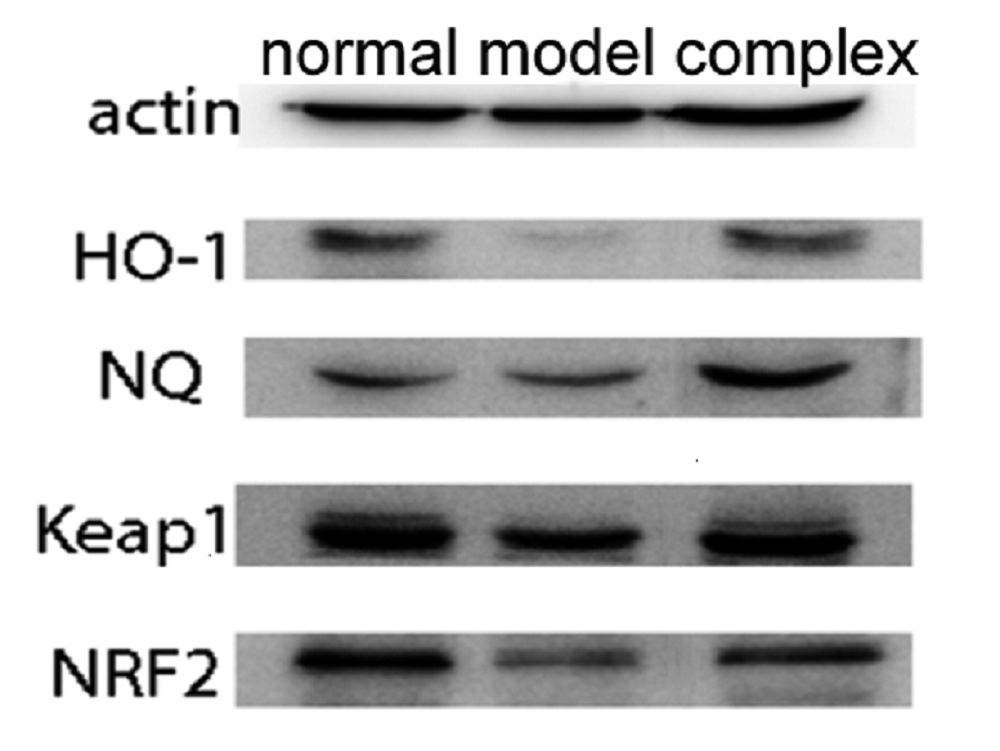
Fig.9

Supplement: supplementary.docx [file IDRD_A_1616236_SM1488.docx]
